# The Wisdom Acquired During Emergencies Scale – Development and Validity

**DOI:** 10.3389/fpsyg.2021.713404

**Published:** 2021-10-07

**Authors:** Giovanni Battista Flebus, Angela Tagini, Marta Minonzio, Eralda Dushku, Franca Crippa

**Affiliations:** Department of Psychology, University of Milano-Bicocca, Milan, Italy

**Keywords:** COVID-19, pandemic lockdown, validation, optimal scoring, wisdom scale, emotion regulation

## Abstract

The COVID-19 pandemic and its related lockdown restrictions had repercussions on health status, psychological states of mind, and emotion regulation. Attitudes towards these restrictions, beliefs, emotions and behaviours could be wise, as in the acceptance of, and adaptation to, these constraints. On the other hand, they could be unwise, as in the rejection of rules and limitations, denial of the consequences, irrational beliefs, self-accusation, rage and general intolerance. This study aims to introduce the development and validation of the 25-item Wisdom Acquired During Emergencies Scale (WADES). It is a measure to assess the wisdom and self-regulation that are needed to cope with unexpected and unpredictable emergency situations. On the basis of a preliminary study (*N_1_*=212 Italian adults), a multiple-choice scale of 52 items was developed. In the reliability study (*N_2_*=1777), items were scaled, analysed according to the optimal score technique and selected to provide a final and reliable version (Cronbach’s *α*=0.83). The validity study (*N_3_*=1,345, *N_4_*=1,445, *N_5_*= 878) provided correlations with established scales measuring, for example, traditional wisdom, emotion regulation, empathy, post-traumatic growth, collectivism, conscientiousness and satisfaction with life. The results confirmed that high scores on the WADES are associated with the ability to regulate emotions, control impulses and develop goals in emotional situations, to tolerate current difficulties, while developing new attitudes, values and behaviours, entailing changes in self-perception and relationships. It was thus confirmed that high WADES scores indicate a higher degree of acquired wisdom.

## Introduction

The coronavirus pandemic of 2019 (COVID-19) entailed a worldwide outbreak of respiratory illness (Zhang et al., 2020). Italy, and the region of Lombardy in particular, was the earliest affected area in the Western world with 243,506 cases and 34,997 deaths reported by the end of July 2020. The Italian Government responded to the outbreak by implementing a country-wide lockdown in March 2020. It is well-known that the COVID-19 pandemic also affected mental health and well-being (Fiorillo and Gorwood, 2020). The related social distancing and self-isolation can impact mental health, also considering that reduced social interactions are well-known risk factors for mental disorders (*ibidem*).

The concept of wisdom has been linked to the ability to recognise and cope with uncertain situations. This construct of wisdom is multifaceted and complex, involving behaviours, attitudes, cognitive abilities and emotional states at both personal and interpersonal levels (Staudinger and Glück, 2011). A number of influential models have attempted to capture the concept of wisdom in terms of subcomponents. Thus, Bangen et al. (2013) suggested that over half of the definitions of wisdom in literature include the following central elements: decision-making related to the social world and pragmatically oriented life-skills; a prosocial stance, including altruism and empathy; an ability to reflect on oneself; emotional regulation; and finally, the ability to acknowledge and react constructively to uncertainty. The latter more contextual aspect is also underlined by other authors (Bigelow, 1992; Kunzmann and Baltes, 2003), who defined wisdom as a type of expertise that operates in undetermined situations, including the ability to tolerate the ambiguity and uncertainty of human existence. In accordance with philosophical traditions and lay beliefs, a number of empirical researchers have also underlined moral aspects of the construct of wisdom (see for example, Sternberg, 1998; Zheng and Wang, 2014; Fowers et al., 2017, 2021). Glück and Bluck’s (2013) MORE model of wisdom suggested that mastery, openness, reflectivity, emotion regulation and empathy are essential aspects of the construct (also see Kunzmann and Glück, 2019). A more recent model suggests that wisdom entails the application of morally informed metacognitive reasoning and problem-solving (Grossman et al., 2020). These authors also underlined the importance of balancing interests of the self and of others, the acknowledgement of a common humanity and a quest for truth. Finally, Sternberg and Karami (2021) suggested a ‘6P model of wisdom’ which includes: (1) *purpose*, for example in terms of seeking the common good; (2) *environmental press* or situations that stimulate wisdom such as the current pandemic, which necessarily imply values of both individuals and groups; (3) characteristics of *wisdom-based problems*, which by definition are ill-defined, contextual, resolved only to varying degrees, require balancing conflictual interests, are often emotional in nature, which are all characteristics of the current pandemic and its consequences; (4) *persons*, i.e., the characteristics of wise individuals which are highly variable; (5) the *process* of wisdom which entails motivational, affective, cognitive, spiritual and metacognitive processes; and finally (6) the *products* of wisdom such as solving problems, decision-making and subsequent actions. However, according to the authors, none of the existing models address all the above-mentioned perspectives.

The clinical literature (Puechlong et al., 2020), lay beliefs and some wisdom researchers (see Glück et al., 2020 for example) also suggest that emotion regulation (ER) may be essential in situations that are perceived as stressful, traumatic or potentially harmful, although this aspect is still somewhat controversial in wisdom research (Grossman et al., 2020). A broad definition of ER entails an unconscious or conscious effort to modulate one’s felt or expressed emotion (Mauss et al., 2007). In situations of emotional distress, ER implies evaluating and changing emotional responses and behaviours (Thompson, 1994; Gratz and Roemer, 2004).

In the past, quarantine measures were associated with widespread fear and uncertainty, the discrimination of certain social groups, economic hardship and social rebellion (Hull, 2005). However, in accordance with resilience theory, the outcome of stressful or traumatic situations is not always harmful. It is of great importance to identify protective factors that can enhance positive responses to adverse events (Marčinko et al., 2020). In line with this framework, the concept of post-traumatic growth (PTG) was proposed (Tedeschi and Calhoun, 1996), which describes positive changes in levels of functioning, lifestyle and the development of insight, in response to highly adverse events. This kind of reaction to negative events seems to be linked to both personal and environmental resources that favour coping strategies (Prati and Pietrantoni, 2009). Three dimensions of PTG were suggested: at a personal level, changes in self-perception including an increase in self-value; at an interpersonal level, higher levels of empathy and closeness to others; and finally, at the level of life philosophy, changes in values and priorities.

Similarly, social scientists distinguish between individualistic societies that value independence and in which personal aims prevail and those based on collectivism. The latter orientation conceives of individuals as interconnected with others, with the group’s well-being and harmony being a priority (Triandis and Gelfand, 2012). In emergency situations, such as pandemics, individual needs may in fact be in conflict with those of the wider community, and thus, a collectivist orientation may constitute a further dimension of situational wisdom during the health crisis. Recent literature (Fowers et al., 2021) focusing on virtues during the COVID-19 pandemic also underlined the importance of finding a balance between individual needs and the collective good, as well as the necessity to take appropriate risks and adhere to safety protocols. The construct of conscientiousness, which captures some of these aspects, refers to a global personality trait characterised by several facets, including industriousness, diligence, dutifulness and perseverance (MacCann et al., 2009). Conscientiousness thus refers to the likelihood of following rules and adhering to regulations, both collectively and personally. Moreover, a growing body of research shows that conscientiousness acts as a protective factor of health (Bogg and Roberts, 2004; Friedman and Kern, 2014; Israel et al., 2014).

The notion of *Acquired wisdom* is defined as an attitude that can develop in individuals who have to come to terms with a potentially life-threatening situation. These events entail being suddenly and unpredictably forced to comply with a number of restrictions such as being confined to one’s home, thus having limited (or forced) personal and/or social contacts. Wisdom entails the need to quickly develop new behaviours, attitudes and psychological strategies to face both the restrictions and fear of contagion. These emotional and cognitive strategies and behaviours may be rated as more or less wise, rational, adaptive, secure, safe and sensible.

As discussed above, wisdom is generally conceived in terms of a long-term attitude or behaviour, while acquired wisdom refers to a temporary state, which includes the ability to refrain from irrational, maladaptive or despairing states of mind. Our construct concerns specific behaviours (e.g., prophylactic actions) and attitudes or emotions (e.g., fear of contagion) as reactions to the realistic threat of dying, being infected or infecting (significant) others. Another difference between our concept and the other models and measures of wisdom is that they do not usually consider disturbing or highly maladaptive thoughts like fear of persecution, fear of insanity or conspiracy theories. Our scale explicitly measures the opposite pole to wisdom and rationality.

From a methodological point of view, there is an inherent difficulty in defining wisdom with self-report Likert scales. The maximum of a scale can only be achieved by endorsing items like *I am wise* or *I always behave wisely* to a high degree. However, authentically wise individuals are unlikely to describe themselves as very wise because they are aware of their limits, while the unwise are more likely to grossly overestimate their wisdom (Glück et al., 2013; Flebus, 2017). One way of avoiding this difficulty is to resort to a multiple-choice format, the same format as the questions used in tests of maximum performance. The problem is that this scoring method is not frequently applied in psychological measurement: it is the optimal scores method, also known as *correspondence analysis* (Benzécri, 1992; Greenacre, 2007) reciprocal averaging, dual scaling (Nishisato, 1980), Guttman’s least squares method (Guttman, 1950) and by a number of other names. This method refers to Eckart and Young’s (1936) theorem of spectral decomposition (see also Weller and Romney, 1990), which is an equivalent of canonical analysis with dummy variables corresponding to the nominal scale (Tanaka, 1979).

By employing this method, the final result is a set of weights to be applied to the items, on a *z*-score scale, which is the same scale as the factor scores (hence the term correspondence analysis). The fact that the method is hardly known does not prevent its use with duly phrased items. As a matter of fact, items should be cast in such a way that the targeted underlying variable can easily emerge as the first dimension, namely, items are formulated so that they are very likely to tap the targeted concept. For instance, in the case of wisdom, the options are either very wise or very unwise, while some options fall in between. The method is still experimental, although some published articles or papers in international conferences (Flebus Flebus, 2017) testify to its appropriateness (Flebus, 1988, 2019). In a self-report questionnaire, there is no correct answer; however, if there is an intrinsically homogenous underlying trait in the items, scaling can still be applied which will yield a set of weights (e.g., −1.3, −1.0, −0.8, 0.5, 0.95) for every item, which correspond to varying degrees with the options of a given item.

### General Aim of the Study

The present studies aimed to develop a questionnaire to measure the acquired wisdom, arising in times of sudden, unforeseen and devastating epidemics, such as the COVID-19 epidemic in Italy during late winter 2020. It was hypothesised that a general psychological trait or dimension (for sake of brevity termed ‘*wisdom’*) exists and can therefore be measured, with an appropriately devised questionnaire. The format is well-known (multiple-choice format) but new in its application. In fact, multiple-choice questions are only used to measure maximum performance traits, because for each question, there is an exact answer, but these are almost never used for typical performance traits, where it is hard (or impossible) to indicate the right answer.

Likert scales are commonly used as closed-ended responses that allow quick extractions and analyses (Baburajan et al., 2020). A widely shared criticism concerns not only the indeterminacy of the number of grades and response styles (Preston and Colman, 2000; Simms et al., 2019), but also the lack of commonly shared guidelines for choosing the rating scales for specific research (Taherdoost, 2019). These limits can introduce serious biases in the measurement of certain constructs such as wisdom. A different approach is possible by asking respondents to express their best, or preferred, option or their first choice (multiple-choice). For instance, the situational judgment tests (SJT) include a situation followed by several possible responses. These responses are nominal multiple-choice options, and their scoring is performed by relying on item response theory-based procedures or on specific methods for nominal responses (Revuelta and Ximénez, 2017; Revuelta et al., 2020; Zu and Kyllonen, 2020).

Unlike Likert scales, in the present questionnaire, each item had several nominal options from which participants selected an answer. There was no *a priori* weight assigned to answers. The self-calibrating score method allowed a transformation of respondent’s choices into numerical measures and subsequently into a measurement of the latent variable. In particular, no pre-existing underlying order was hypothesised for the nominal options, although it may have existed in the minds of respondents (see for example, Revuelta et al., 2020). In summary, Likert’s scales posit *a priori* scores, the values are recognisable, and the scales have an order from low to high. In the optimal scoring, only *a posteriori* scores are available.

Another objective was to illustrate a novel procedure used to develop a questionnaire, with a different method of scaling items. The suggested procedure, described in detail below, is particularly useful because the main concept (acquired wisdom) is highly complex, with a variety of facets that would require an extremely high number of items or scales to provide an acceptable measurement.

### Phase 1: Questionnaire Development

#### Aim

The aim was to develop a new questionnaire to measure the construct of acquired wisdom. Given the novelty of the epidemic and the related lockdown, it was judged that research from scratch was needed.

#### Participants

A snowball technique was used to contact the participants online. The questions were distributed to Italian-speaking participants, with the help of psychology students who received academic credits for their participation. The instructions asked participants to complete the provided sentences with personal views, thoughts and feelings.

Respondents were 212 Italians (sample N*_1_*), of diverse social demographic backgrounds (aged from 19 to 87, 72% females, 21% from Lombardy, 49% with a university degree).

#### Methods

##### Procedure

A written sentence completion technique, drawing from Nuttin’s Methode d’Induction Motivationnelle (Nuttin, 1985) or the 40 Complete sentence list (Rotter and Rafferty, 1950), was adopted. This technique is used both in clinical practice and research (Holaday et al., 2000) and is very efficient in obtaining and accumulating new material and suggestions for research. Sixty-three sentences to be completed by participants were administered (see Supplementary Material). Examples of these sentences are *Once the quarantine is over, I am afraid/I hope*… or *At the moment, what is the most difficult to bear is*… and: *Despite the health crisis, people do not seem to understand that*…

#### Results

The complete answers were 142. The research team formulated 91 items which underwent scrutiny as to readability, content and strict adherence to the aims of the research. All items had to offer one or two very wise options and one or two extremely unwise options. The initial form for each item included 8 to 10 options to facilitate a final quick selection. Unwise options could refer to any form of lack of wisdom, namely, excess of anger, persecutory thoughts, regrets, excessive concerns for one’s health, despair, depression, extreme individualism, perfectionism or extreme control. At the end of the collective scrutiny, 52 items were selected that had the same format: a stem sentence to be completed with an answer chosen from seven options. An example of an item and the seven possible answers is the following: I will be happy when…(1) They will find a vaccine and infections will diminish; (2) Those who are not law-abiding will be punished; (3) Those who are responsible for the pandemic will be punished; (4) I will no longer be forced to be alone; (5) People will admit it was all a joke; (6) I will be able to escape from this house or this city; (7) I will be free to do whatever I wish.

### Phase 2: Item Selection and Reliability

#### Aim

This study aimed to select the items of the WADES questionnaire. In this process, we merged the assessment of the internal validity of the questionnaire.

#### Participants

The research questions were presented randomly after a number of socio-demographic questions. The questionnaires were distributed online to 2,233 Italian-speaking participants (living in or outside of Italy), with the help of psychology students who received academic credits for their participation. The usable sample (criterion: at least 26 questions completed) was made of 1777 individuals (sample N*_2_*), aged from 17 to 86years (*M*=31.15, *SD*=15.05), mostly highly educated (38.9% had a university-level education) and mostly from Lombardy (56.5%).

#### Methods

##### Procedure

The 52 nominal items could not be submitted to a regular factor analysis. Categorical factor analysis, also known as correspondence analysis, was thus performed. The Categorical Principal Component Analysis (CatPCA) Subroutine of SPSS computes (a) the weights for all options for all items (b) scores for all participants and (c) Cronbach *α* coefficient (the default is two dimensions or factors but we only requested one). For each item, the subroutine computes a discriminant index which can be used to discard it, if the correlation with the factor is low; Cronbach’s *α* allows for a reliability analysis (if a bad item is discarded, the alpha coefficient rises and confirms that the discarding was legitimate).

Initially, the sample was split into two subsamples (A and B). The subsample A was used to locate poor variables, namely, those which did not reach at least 11% of common variance with the first factor (equivalent to a factor loading of 0.31). After discarding low discriminating items, the analysis was cross-validated on subsample B either to confirm the good items or to remove further ill-suited items (17 items were thus eliminated).

The process of item selection underwent a second step by means of a search for correlated residuals (Hambleton and Rovinelli, 1986). To perform the latter, all items were recoded with their numerical weights obtained by means of CatPCA and submitted to regular factor analysis, without rotation with seven factors being extracted (the number was set arbitrarily). If high secondary loadings (cross-loadings) emerged (i.e., there were correlated errors among certain variables), the offending items were discarded one at a time and the analysis was repeated until no more offending items emerged (Lord and Novick, 1968). This kind of item analysis left us with 30 items. However, there was still room for further selection, because 30 items were deemed too many for a one-dimensional scale. Thus, we took into account the composition of the whole sample, composed of 34.8%, males and 65.2% females, 50.3% up to 22years and 49.7% from 23years to 87years, 61.2% up to high school and 38.9% with a university degree. The sample was stratified – one variable at a time – according to gender, age and education. On each subsample, a regular exploratory factor analysis (performed on the CatPCA outcome) located those items with high residual loadings on factors beyond the first. Five further items were thus discarded.

The 25 questions of the final questionnaire are too long to report here (for the full version please see the Supplementary Materials, Suppl. 2). The weights are *z*-scores, which testify the position of each option along the continuum from low (negative scores) to high wisdom (positive scores). The administration of the questionnaire is relatively simple: the questionnaire, composed of 25 questions with 7 options each (totalling 175 answers) is administered. Each chosen answer is then recoded with its quantification (presented in the Supplementary Materials).

Finally, because the scores – expressed as *z*-scores – did not have a fully Gaussian shape, they were normalised and slightly adjusted to follow a really normal distribution (subroutine RANK of SPSS).

### Results

The final 25 items all moderately loaded on the first factor (*min* 0.307, *max* 0.496, *mean* and *median*=0.397; see the suppl4 in Supplementary Materials for the whole factor matrix), with an alpha coefficient of 0.827, with no strong bias related to age, gender and education. Table 1 presents the final weights for two items as an example.

**Table 1:** Two examples of multiple-choice items.

Item		frequency	weight
**# 9: At bedtime…**	I feel overwhelmed by anxiety	194	−0.802
	I spend a lot of time online	1,241	−0.701
	I wonder for how long I will be able to bear this situation	400	−0.694
	I constantly think about the current situation	177	−0.004
	I feel better thinking about my loved ones	1,418	0.223
	I prepare for tomorrow, setting myself some goals	1888	0.369
	I think that sooner or later this situation will end	546	0.502
**# 25: This period will teach us all…**	That no one can save us	34	−1.755
	That one cannot trust people	47	−1.477
	Nothing, people do not change	1,351	−0.749
	That the world is not a safe place	71	−0.450
	To show more solidarity and be less selfish	752	0.146
	To be more conscientious and have more common sense	1715	0.261
	To recognise our limits and that we are not invincible	1935	0.326

**Figure 1:**
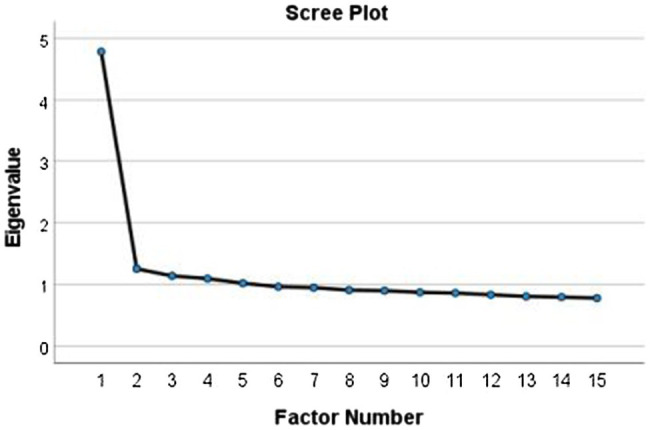
Scree test of the first 15 eigenvalues extracted from the matrix of the 25 optimally scaled items.

Figure 1 displays the scree plot for the 25 items, which suggests that there is only one factor in the data, as expected with efficient and reliable one-dimensional scales. One large eigenvalue, extracted from a 25-item correlation matrix and negligible succeeding eigenvalues, testify that the effort to assign numerical coding to each of the options of the 25 items as a quantification was a legitimate procedure.

**Figure 2:**
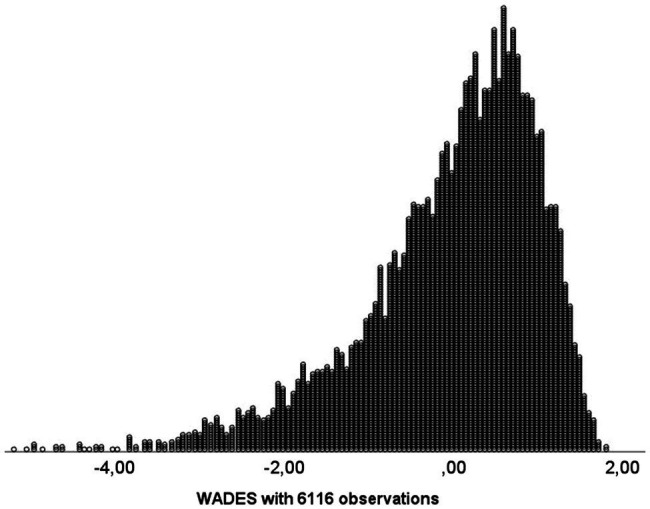
Distribution frequency of the optimally scaled scores (*N*=6,116).

**Figure 3:**
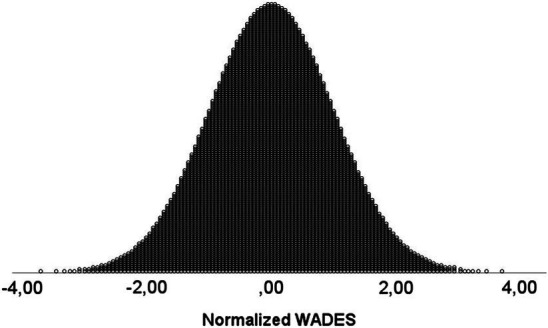
Smoothed scores.

Figures 2, 3 present the scores before and after the normalisation. The Bravais-Pearson correlation between the two measures was 0.955, whereas Kendall’s *tau* and Spearman correlations were exactly 1.00.

The average of the 25-item scores is computed and then divided by the standard deviation (*s.d*. = 0.40241). The result of this computation can be considered a z-score and interpreted as such. A high score signifies high levels of wisdom, while a low score suggests low levels of wisdom. In order to convert the z-scores into percentiles, the reader can refer to Suppl3 in the Supplementary Materials.

### Phase 3: Concurrent Validation

#### Aim

This study aimed to determine the concurrent validity of the wisdom scale. To this end, the WADES scores were compared with established scales, measuring a number of variables which have previously been associated with the general construct of wisdom or that were hypothesised to be particularly relevant to the current pandemic: (1) general wisdom (2) the ability to regulate emotions and behaviours (3) empathy (4) a sense of community and common good (5) conscientiousness and the ability to follow rules (6) a capacity of post-traumatic growth (7) satisfaction with life and (8) proneness to describe oneself with a questionnaire in a non-conflictual and limpid way (vs. mental reservation or reluctance). Finally, it was hypothesised that *respondability* (i.e., relevance, clarity and lack of reticence) would be lower for those respondents who are critical about lockdown or provide unwise answers: conflictual, contrasting harsh feelings or mixed feelings about lockdown and its rules were hypothesised to conflict with a clear sense of self, which entails cognitive-affective strategies to avoid feeling of uneasiness, both towards lockdown and the questionnaire that investigates reactions to the restrictions.

#### Participants

Three different samples were recruited online (*N_3_*=1756, *N_4_*=1,234; *N_5_*=850) with demographic characteristics comparable to the sample N*_2_* of Phase 2.

#### Method

##### Measures

The following questionnaires were administered:

*Brief Wisdom Screening Scale*. Glück et al. (2013) developed this scale empirically by selecting the items from three self-report measures, each aimed at measuring different facets of the wisdom construct. However, only the first 16 items were selected (which loaded highly on the first wisdom dimension). Its reliability was adequate (Cronbach’s *α*=0.78, it was 0.84 in the original version of 20 items). An example of the items is: ‘*I am able to integrate the different aspects of my life*…’.

*Difficulties in Emotion Regulation Scale*. (Gratz and Roemer, 2004) The Italian version was administered to evaluate difficulties in emotion regulation. This scale consists of six clinically relevant aspects of a lack of emotion regulation: A lack of acceptance of emotional responses (Non-acceptance); a lack of emotional awareness (Awareness); a limited range of emotion regulation strategies (Strategies); difficulties in goal-directed behaviour when emotional (Goals); impulse control difficulties (Impulse); and a lack of emotional clarity (Clarity). The Italian scale has excellent reliability (Cronbach’s *α*=0.92, Giromini et al., 2012) for the total DERS score. An item example is: ‘*When I’m upset, I acknowledge my emotions*’.

*The Interpersonal Reactivity Index*. The Italian version of the Interpersonal Reactivity Index (IRI; Davis, 1980; Albiero et al., 2006) assesses empathy as a multidimensional construct, in terms of social functioning, self-esteem, emotionality and sensitivity to others by means of 28 items divided into 4 subscales: Perspective Taking, as the ability to adopt the point of view of others; Empathic Concern, which measures concern and sympathy with others perceived to be unfortunate; Personal Distress assesses anxiety and uneasiness in difficult interpersonal situations. Internal consistency is good with alpha coefficients varying between 0.63 and 0.74. The Fantasy scale was deemed irrelevant to the pandemic situation and thus omitted. An item example is: ‘*I am usually pretty effective in dealing with emergencies*’.

*Concise Scale of Individualism–Collectivism*. By developing this scale, Chen et al. (2015) meant to explore the relationship between culture and health. Nine of the 18 items investigate the level of individualism and the other nine the level of collectivism. Owing to the length of the scale, we decided to trim it down to only four items (two related to individualism and two to collectivism). An example is ‘*A person must follow only his/her own ideas in relation to determining the best actions and behaviour*.’

*Conscientiousness*. We used eight items from the (Goldberg, 1999; International Personality Item Pool- IPIP, 2001; Goldberg et al., 2006). Five items refer to scrupulous and responsible behaviours: ‘*I make plans and stick to them*’, The remaining three items refer to irresponsible actions: ‘*I waste my time*’ and ‘*I escape my duties*’.

*Post-traumatic Growth Inventory*. The Italian version of the Post-traumatic Growth Inventory (PTGI; Tedeschi and Calhoun, 1996; Prati and Pietrantoni, 2014) is a 21-item scale that measures positive changes in individuals who experienced adverse events. The scores refer to five different dimensions: Relationships with others, New possibilities or purposes, Appreciation of life, Spiritual changes and Personal strength, rated on Likert scales ranging from 0 (no change) to 5 (high levels of change). The scale has an excellent internal consistency (Cronbach’s *α*=0.93). In the present study, participants were asked to refer to the lockdown condition due to the COVID-19 pandemic as the adverse event. An item example is: ‘*I changed my priorities about what is important in life’*.

*Satisfaction With Life Scale*. The subjective perception of personal well-being comprises an emotional and a cognitive dimension (Diener, 1984). Satisfaction with one’s life belongs to the cognitive sphere of personal well-being (Andrews and Withey, 1976). In this regard, Diener et al. (1985) proposed a five-item scale for the measurement of satisfaction for life in general, high scores reflect a strong appreciation of life and the impression that things are going well (Mead, 2020). On the contrary, extremely low scores suggest dissatisfaction with the present circumstances (Pavot and Diener, 2013). Cronbach’s *α* of this scale is 0.83. An item example is: ‘*My life is close to the ideal one under several points of view’*.

*The Questionnaire to Measure Attitudes towards a Questionnaire*. (Flebus, 2007) It is a one-dimensional measurement (‘Respondability’) composed of three facets: Reticence (in describing oneself), Clarity (of the meaning and the items) and Relevance (or curiosity and interest in the topic raised by the questionnaire). The current version has a good reliability (Cronbach’s *α*=0.78) Example: ‘*I tried to respond as accurately as possible’*.

### Results

Convergent validation consisted of computing Bravais-Pearson correlation coefficients between the WADES score (computed as the sum of the 25 weights) and the various other scales administered in this study (Table 2).

**Table 2:** Pearson correlation of scales with WADES.

*Scale*	*r*
**Brief Wisdom Screening Scale**	0.22^***^
**DERS (Difficulties in Emotion Regulation Scale)**	
Limited access to emotion regulation strategies	−0.37^***^
Lack of emotional clarity	−0.34^***^
Impulse control difficulties	−0.31^***^
Difficulty engaging in Goal-directed behaviour	−0.29^***^
Lack of emotional awareness	−0.18^***^
Non-acceptance of emotional responses	−0.17^***^
Total score	−0.39^***^
**IRI (Interpersonal Reactivity Index)**	
Personal distress	−0.08^*^
Empathic Concern	0.40^***^
Perspective Taking	0.20^***^
**CSIC (Concise Scale of Individualism–Collectivism)**	0.18^***^
**Conscientiousness**	0.33^***^
**PTGI (Post-traumatic Growth Inventory)**	
Relating to others	0.30^***^
Appreciation of life	0.32^***^
Personal strength	0.23^***^
New possibilities	0.22^***^
Spiritual change	0.10^**^
Total score	0.30^***^
**SWLS (Satisfaction With Life Scale)**	0.29^***^
**QUAQ (attitude towards questionnaires scale)**	0.14^***^

* p<0.05; ^**^p<0.01;

*** *p<0.001*.

With reference to the single scales as shown in Table 2, WADES proved positively correlated with the concise wisdom scale by Glück *(r*=0.22, *p*<0.001), suggesting a limited overlap with the construct of wisdom.

Negative correlations between the WADES and all the subscales of the Difficulties in Emotional Regulation Scale were found (ranging from −0.18 to −0.37). This suggests that individuals with high levels of wisdom had fewer difficulties in regulating emotions, achieving emotional clarity, controlling their impulses and engaging in goal-directed behaviours. The correlation with the total score was moderate (*r* =−0.39, *p*<0.001).

The relation of the WADES with the IRI scales indicated that participants with high levels of personal distress had lower scores of wisdom, although the effect size was very small, accounting for less than 1 per cent of common variance. Empathic concern for others had the highest positive correlation with the WADES, while the ability to assume others’ perspectives was also associated with wise attitudes to the pandemic, even though to a lesser extent.

The correlation between the WADES and Collectivism was significant but rather low (*r* =0.18, *p* <0.001), while the association with Conscientiousness was positive and moderate (*r*=0.33, *p*<0.001).

The association with Post-Traumatic Growth (PTG) corroborated the concept of situational wisdom during an emergency. All the PTG dimensions were positively correlated with WADES, with the highest correlation value in correspondence to Appreciation of life (*r*=0.32, *p*<0.001), followed by Changes in relations with others (*r*=0.30, *p*<0.001), Personal strength (*r*=0.23, *p*<0.001) and New possibilities (*r*=0.22, *p*<0.001), while Spiritual change only reached a value of 0.10 (*p*<0.0001). The WADES correlation with the total score of the PTG was also moderate (*r*=0.30, *p*<0.001).

The correlation with Satisfaction with life (*r*=0.29, *p*<0.001) also confirmed the initial hypothesis. Finally, the low correlation with the Attitude towards Questionnaires Scale marginally supported the concurrent validation with modest values, equal to 0.14 (*p*<0.001).

As far as socio-demographic variables are concerned, a significant effect size was found for gender (*η^2^*=0.025, women were wiser), education level (*η^2^*=0.013, situational acquired wisdom was higher in more educated individuals) and age (*R^2^*=0.096, older people were wiser). Individuals who felt at risk of contagion (on a 1 to 7 scale) were wiser (*η^2^*=0.059), religious individuals (*η^2^*=0.103) and those who were impacted in their professional life (*η^2^*=0.013) had more situational wisdom; the region of origin also had a small size effect (*η^2^*=0.021).

### General Discussion

This study aimed to develop a new questionnaire in order to evaluate the situational or acquired wisdom arising during a collective emergency like the COVID-19 pandemic. We hypothesised that the construct is multifaceted and that it can be measured. We suggested that the easiest way to describe acquired wisdom is to report the sentences (and relative weights) that wise individuals chose to complete multiple-choice questions regarding the pandemic and lockdown.

In contrast with the maximum performance questions, in which exact responses are quantified with 1 and the inexact ones with zero, the novelty of the present scale is that responses were *unspecified, and therefore, there were no right or wrong responses. However*, an underlying continuum was hypothesised, with options ranging from low to high on a continuum of wisdom, rationality and appropriateness. The numerical weight (to be assigned to an option) was therefore not known *a priori*. Instead, the weight emerged from a comparison among the responses of all participants; individuals who were wise, rational and sensible chose wise, rational and sensible responses, and their scores helped to determine the weight for each option. The iterative process always reaches the same final result, independently from the initial hypothetical start (this is also called Guttman’s Least squares method, 1950). One can wonder if the wisdom score is always appropriate and rationally founded. The statistical method prevents misattributions, as long as we assume that the majority of the items covers the specified domain. At most, the risk is that a multiple-choice question has a low discriminant value (weights for all options are very similar). If that is the case, the item can be easily eliminated (see also Flebus, 1988 for a similar application). In fact, we discarded 17 items because the options did not discriminate among respondents, their weights were too similar and their contribution to the reliability of the questionnaire was too small.

It should also be noted that the number of participants who chose a certain option did not necessarily determine the numerical value of the weight: an extreme score was associated with a small group, but a small group does not inevitably entail having an extreme score.

Question 9 (see Table 1) exemplifies the results: for the question At bedtime, 194 individuals chose the option *I feel overwhelmed by anxiety*, whose weight was −0.802. For that option, this was also the mean of the 194 participants on the WADES score. A less extreme option was *I spend a lot of time online*, chosen by 1,241 individuals, with a weight of −0.701. The highest weight was 0.502, denoting the wisest answer (*I feel better thinking about my loved ones*) among the seven possible options (546 respondents).

The second item’s stem refers to the current historical moment: #25. This period will teach us…34 participants chose the extreme, pessimistic answer… *That no one can save us*. This small number of despairing individuals had a very low mean weight (−1.755). The following group of responses was slightly more rational, with 47 individuals totalising a score of −1.477. The subsequent group was quite numerous, 1,351, with a score of −0.749. The last group, which had the wisest response, with a mean weight of 0.326, was also the most numerous, 1935. Note that we did not find very high positive weights, the extreme values were only negative. This is due to the fact that wisdom is also a common, widely shared point of view, for which a consensus can be found. In contrast, unwise attitudes and behaviours can be more diverse in nature, for example, individuals can be highly irrational, vulnerable or defiant.

Wise individuals chose wise responses, which in turn could be used to define wisdom, the following being some examples (abridged citations from the items): The situation is … *Difficult, but bearable* (0.38). For kids, being isolated with their parents, the lockdown … *Is an opportunity to know each other better* (0.64), or for kids, being isolated with their parents, the lockdown *is just great* (0.51). For their parents, it is … *An opportunity to share thoughts and rediscover values* (0.53). Beliefs … *Are helpful if they concern ethical principles and science* (0.39). Wise individuals would comment on others’ losses saying … *We need to share the pain and memorie*s (0.38). The most often felt feelings are … *Concerns about the health of my loved ones* (0.47) or *A sense of solidarity and communion with others* (0.52). They will feel … *Hopeful, that this health crisis will be resolved* (0.40) and are reassured that … *My family and I are adopting all precautionary measures* (0.37). The most positive aspect of this period is … *Rediscovering values like the solidarity among people* (0.44). However, the wise feel sad when they think … *About the victims of the epidemic and their family and friends* (0.43) and find that The most distressing thought is … that *My loved ones or I can get ill* (0.38).

On the other hand, the most irrational, fearful or vulnerable respondents chose different options. I will be happy… *When people will admit it was all a joke* (−1.78) or when *I will be able to escape from this house or this city* (−1.20). They are convinced that … *People deliberately infect others* (−1.32), or *Do not care about people* (−2.48). What is most needed in this situation is… *Being able to vent and distract oneself* (−1.28). For kids, being isolated with their parents … *Is the most stressing thing at the moment* (−1.42) or *Can be sheer torture* (−1.52), whereas For parents, quarantine … *Is exhausting* (−1.49). This is the way they would make a comment To those who lost a loved one … *The lost of your loved one is just one among many others* (−1.82) or *You need to see a psychic* (−1.88).

One may wonder why the latter common sense-statement received such low weights. Although this is not an irrational statement, within the given options, it appealed to the least wise people, perhaps because it is a cliché. The general rule of the multiple-choice questions applies here: all items receive a certain weight not because they depict a wise (or unwise) response which is wise (or unwise) in itself, but because it was selected by wise (or unwise) individuals among the other available options.

Participants were most convinced that What was needed during lockdown was the belief that … *A solution would be found* (0.16) followed by *Empathy and emotional support* (0.20) and *Distractions* (−1.28). The best thing about having to stay home is … *Being able to avoid the people we dislike* (−1.30) or *Nothing* (−1.17). And further, The most positive aspect of this period is either … *Nothing positive* (−1.17), or *That they are not forced to see people* (−1.20) or *Being able to sleep* or *do absolutely nothing* (−1.43).

On the basis of our results, we propose that a wise, rational and sensible attitude towards quarantine and restrictions is a psychological dimension and that its measurement responds to the current requirements for test development. The high Cronbach alpha (0.83) suggests that the WADES is in fact a reliable measurement.

The final scale considers a number of different ways of conceiving the outcomes of pandemics and lockdowns: (1) emotional self-descriptions (#1 I’ll be happy when… #10 The feelings I have had more often are…#11 When I think of the current situation, I feel… #14 The thought that I find most distressing during this period is… #16 I’m reassured by the fact that… #18 I’m very sad when I think…); (2) emotional reactions to the lockdown and to potential infections (item s09 At bedtime…#15 The best thing about having to stay home is…#19 During this crisis I ended up…); (3) behaviours (#22 The most useful thing I have done…); (4) attitudes (#13 During this period, what makes me feel better is…); (5) coping mechanisms (#20 In order to calm myself down, I…); (6) Social aspects (#02 I’m convinced that most people…#03 I’m afraid that people… #12 The most unacceptable behaviours I heard about were…); (7) Empathy or general concern for others (#04 Above all, during this period what’s needed is…#08 To those who lost a loved one in this situation, I would like to say that…); (8) The significance of close relationships (#05 For kids, being isolated with their parents…) #06 (For those who have children, being quarantined…#17 I would like the people I live with to…) as well as for neighbours (#23 When I think of my neighbours…); (9) aspects of conscientiousness and personal growth (#24 Apart from following the regulations established by authorities, it would be helpful if one could…#25 This period will teach us…#21 The most positive aspect of this period is…); and (10) Attitudes to religious beliefs: #07 At the moment I think beliefs… The full table of the 175 options should be kept at hand for a thorough comprehension of the scale.

As to the validity of the WADES, the significant correlations with the questionnaires confirm the original hypotheses. In other words, this type of wisdom entails the ability to (1) regulate emotions, consider the perspective of others, empathise with both other individuals and the wider community (2) respect temporary limitations imposed in order to diminish contagion and (3) limit personal wishes for the collective good.

Comparisons with existing measures of conscientiousness, emotion regulation, a sense of collectivism and post-traumatic growth indicated that high scores on these measures were in fact related to an ability to cope and adapt to the government-imposed limitations during the COVID-19 emergency. On the contrary, low scores on the WADES were associated with emotional dysregulation (5. When I think of the current situation, I feel … *Helpless and vulnerable*), irrationality (17. Apart from following the regulations established by authorities, it would be helpful if you could… *No longer listen to the news*), persecutory-like ideation (6. When I think of my neighbours… *I fear that they could be a source of infection*), self-accusations or paranoid attributions (18. I am convinced that most people… *Deliberately infect others*) or excess of power (21. The most unacceptable behaviour I have heard about was… *Extremely controlling behaviours on behalf of the police force*) or even *hopelessness* (3. The most positive aspect of this period is…*There is nothing positive about this nightmare*).

The multidimensional composition of the WADES construct appears to be in line with the complexity of past and current accounts of wisdom (Sternberg and Karami, 2021). In particular, concurrent validity represented a central aspect of our study and the results support the hypotheses on which the new scale was based. In fact, our WADES construct implies a spectrum of behavioural and personal responses to a previously unheard-of collective emergency. There is only a limited overlap with the concise scale by Glück which substantiates these aspects. Thus, our WADES scale is not an equivalent measure of the existing perspectives on wisdom, perhaps because of the unexpected effects of the COVID-19 pandemic. As already mentioned, our construct of acquired wisdom differs significantly from Glück’s concept. The WADES intends to measure a temporary state of mind that is induced by an emergency situation. Our construct is more encompassing than the notion of wisdom since it includes the ability to react rationally and adaptively to an unforeseen threat to the individual and the community as a whole. Furthermore, the WADES considers disturbing thoughts and emotions and maladaptive behaviours as well as positive and constructive ones. This view is in line with Fowers et al. (2021) who, from the perspective of practical wisdom, underline that during the COVID-19 pandemic certain fears seem to be ‘wiser’ or more adaptive than others. For example, fears of death or infection may be considered to be wiser than the fear *that the quarantine will make us go insane* (Question 14, option 5) or *that the virus will destroy mankind* (Question 14, option 4) or *that in the future there will be less freedom* (Question 14, option 2). Thus, the WADES format allows for comparisons between *appropriate* and *inappropriate fears*.

Moreover, the WADES refers to dramatic and painful situations, alluded to or explicitly stated in the questionnaire for instance*, The infections are decreasing* (Question 21, option 7) or question 18: I am very sad when I think… *that the ill are dying alone in isolation*. No existing questionnaires referred to such extreme instances of life events when measuring wisdom.

Another difference is the multiple-choice format which allows comparisons between concepts that can be particularly difficult to evaluate, like fears or disturbing behaviours item 3 I am afraid that people… *are going to start breaking the law and will be dangerous* (weight−0.47) vs. *will continue to get ill* (weight 0.15) or *have not understood how serious the situation is* (weight 0.22) and item 14 (The thought that I find most distressing during this period is…) or (Item 12: The most unacceptable behaviours I heard about were…).

On the other hand, the results relative to validity also suggest attitudes and personal perspectives that empower individuals when facing difficult circumstances. The positive correlations between WADES and the Satisfaction with Life Scale (SWLS) indicate that high levels of satisfaction with life and of self-acceptance enable individuals to cope with restrictions and to keep impulses at bay. Moreover, the positive relation between the WADES and the individualism–collectivism scale indicates that situational wisdom also implies being attentive to collective demands, even when these are in conflict with an individual’s need of freedom from lockdown restraints. A similar positive relationship with the Conscientiousness scale further suggests that, as hypothesised, the ability to abide with anti-contagion prophylaxis, to respect imposed regulations and to plan one’s life accordingly also play a part in situational wisdom. The same can be said about the IRI results and, in particular, in relation to empathy and taking others’ perspectives. In fact, unexpected difficult times require the ability to understand how other individuals and the community are affected by the pandemic.

Pronounced self-regulation scores characterised those people who can keep their emotions and impulses at bay and are also capable of controlling their behavioural reactions. This result had also been hypothesised and is congruent with literature about wisdom and its constructive reactions to stress. The regulation of emotion is in fact widely taken into account in wisdom models (e.g., Kunzmann and Glück, 2019). The results confirmed our initial hypothesis, with high scores on the WADES being associated with the ability to accept and be aware of emotions, the ability to implement emotion regulation strategies, control impulses and develop goals in emotional situations. The relation between the WADES and the Difficulties in Emotion Regulation Scale (DERS) of the present study is in line with those found when measuring the cognitive and affective components of the three-dimensional wisdom scale (García-Campayo et al., 2018).

The association with the Post-Traumatic Growth Questionnaire corroborated our concept of situational wisdom during a collective emergency, conceived as the ability to endure current difficulties, while developing new attitudes, values and behaviours, entailing changes in self-perception and in relationships. However, constructive reactions to the pandemic lockdown were less associated with spiritual changes. Those who had difficulties in adapting suffered the most, because they seemed to struggle with finding solutions to the imposed restrictions. Finally, the low correlation with Questionnaire to Measure Attitudes towards a Questionnaire marginally supported the concurrent validation: it can be expected that answering questions on difficult topics like the pandemic and its consequences may cause uneasiness, a defensive lack of coherence and an apparent lack of interest in the issue.

In the future, and considering that it is likely that pandemics will re-occur, the questionnaire may be helpful in identifying individuals who are particularly vulnerable, inclined to respond to these situations in a non-adaptive way, as for example, those respondents who fall in the inferior quartile (below a score of −0.67). Furthermore, an evaluation of the 175 situations summarised by the 25 items could be of interest to policy makers in order to improve interventions and increase social and individual well-being.

## Conclusion

A new construct (acquired or situational wisdom) was measured with a little-known scaling technique. This is a particular application of the optimal scoring method to a one-dimensional trait. Our analyses were largely exploratory because of the lack of previous data or studies on this unprecedented situation. We suggested the term ‘self-calibrating scores’, to emphasise the special situation in which nominal variables are scaled to measure an underlying trait, in which wise individuals are identified by wise answers, while unwise individuals are characterised by unwise responses. The sample size (a very large size for all studies), which supported the new procedure, was also a strong point. Another strength of this study was the high reliability found for the new instrument across several samples.

It should, however, be noted that the unexpected pandemic required a highly specific design of WADES, including some items which may be time and context-sensitive, such as ‘I will be happy … *when a vaccine will be available*’.

A further limit of the study is that a high number of participants were mainly located in one particular region of Italy, namely, Lombardy. However, the latter region was also the one that experienced the worst initial impact of the epidemic in the western world. Another limitation was the age distribution with two modes, one corresponding to age 22 and a second to the age of their parents, around 55years. Although we cannot speculate as to how this distribution may have affected the questionnaire structure, we believe that potential distortions were limited and irrelevant to the validity of the questionnaire.

## Data Availability Statement

The datasets presented in this study can be found in online repositories. The names of the repository/repositories and accession number(s) can be found in the Supplementary Material.

## Ethics Statement

The studies involving human participants were reviewed and approved by the Ethics Committee of the University of Milano-Bicocca. The patients/participants provided their written informed consent to participate in this study.

## Author Contributions

GF: project design and conception, data analysis, writing and critical revision of the manuscript. AT and FC: project design and conception, writing and critical revision of the manuscript. MM: data collection and writing. ED: data collection and writing. All authors contributed to the article and approved the submitted version.

## Funding

This work was supported by the Fund of the University of Milano-Bicocca, Departmental Share (Grant no. 34735, 2020-ATE-0423).

## Conflict of Interest

The authors declare that the research was conducted in the absence of any commercial or financial relationships that could be construed as a potential conflict of interest.

## Publisher’s Note

All claims expressed in this article are solely those of the authors and do not necessarily represent those of their affiliated organizations, or those of the publisher, the editors and the reviewers. Any product that may be evaluated in this article, or claim that may be made by its manufacturer, is not guaranteed or endorsed by the publisher.
