# Supplementary material for: The Wisdom Acquired During Emergencies Scale – Development and Validity
Source: Front Psychol. 2021 Oct 7;12:713404. doi: 10.3389/fpsyg.2021.713404 (PMC8530154; doi:10.3389/fpsyg.2021.713404)
Supplement: Supplementary file 1 [file Table_1.pdf]

Supplement 1: incomplete sentences used in Phase 1 (preliminary study)

Sentence completion

The sentence completion technique requires the respondent to make a whole sentence, starting from a cue. To write a meaningful sentence, the respondents need to search in their memories and experience., therefore revealing their thoughts and emotions in an understandable way. This is considered an equivalent of projection techniques, with evident drawbacks such as a lot of verbal material to be organized by the clinician/researcher. However, it does allow an in-depth exploration of the participants' inner worlds. As a personality assessment instrument, it can still be used for idiographic analysis. As a nomothetic instrument, it requires a thorough psychometric analysis.

Nuttin used Rotter's completion test to develop his MIM, which is used mainly to produce idiographic material. The author suggested modifying the sentences with qualifiers referring to the object of study. So, for a sentence like *I would like in particular*, a modified version to study groups of people would be *As a student/retired / lesbian /inmate. I would like in particular...* In our case, we added complements like **After the epidemic**. *I would like...* Or *What I fear most* ***during the epidemic...*** We also omitted some items, in either Rotter's or Nuttin's list that were not suitable for our purpose.

This list comprises 63 sentences that were used in the sentence completion task. The 142 respondents provided the responses from which we devised the 52 initial multiple-choice items.

1	To those who lost a loved one in this situation I'd like to say…
2	At bed-time….
3	After the quarantine, I hope that …
4	After the quarantine, I'm afraid that…
5	After the emergency, I no longer want to waste my time…
6	After the emergency, my priorities will be…
7	The most absurd thought I had during the quarantine period was….
8	The strangest thing that happened to me during the quarantine period was…
9	During the quarantine I tried to…
10	The most frequent feelings I had were…
11	I'm afraid that people...
12	I'm afraid that the information on the health situation...
13	The most absurd behaviour I witnessed was...
14	The thought that all of Italy is quarantined...
15	The thought that fellow citizens don't have enough money or have lost their jobs...
16	During this period, attempting to have constant updates...
17	In this situation it would not be at all wise …
18	In this situation I'd like to know...
19	At the moment it is unacceptable…

20	At the moment I really need...
21	At the moment I think that beliefs …
22	At the moment, my greatest hope is …
23	During this period, I'm happy when/if...
24	The most distressing thought I had during this period was...
25	The best thing about being limited to one's home is...
26	The most upsetting phrase I heard or read was...
27	People have a strong need for …
28	The main difficulty during this period is...
29	The most positive aspect of this period is...
30	The outdoor activity I miss the most is...
31	The wisest thing I did was...
32	The wisest act I heard about in the media was...
33	The consequences of the quarantine are less severe for...
34	I'm very comforted by the thought of/that …
35	I'm bothered by...
36	It bothers me when my cohabitants…
37	I'd like my neighbours...
38	I'd like for my loved ones...
39	My greatest regret is...
40	I'm very sad about…
41	I've never told anyone that during this emergency...
42	Despite the health emergency, people don't seem to understand that...
43	For those who are alone, being limited to one's home …
44	For children, being on lockdown with their parents …
45	For couples, being limited to one's home…
46	To keep one's head screwed o, I think that...
47	To calm myself down I...
48	What I miss the least is...
49	What I miss the most is...
50	This period will teach us all...
51	I'd be surprised to find out that...
52	I'll be happy when...
53	I think that in 6 months' time the world situation will be...
54	I think that in 6 months' time my family situation will be...
55	I think that in 6 months' time the situation in Italy will be...
56	During this period, only a madman could …
57	I fear that my loved ones...
58	During this period, a rational person with a lot of common sense will avoid …
59	A foolish concern is...
60	After the emergency I'd like relationships between people …
61	For those who have children being limited to one's home...
62	Apart from being infected, people fear that…
63	I think that at the moment religion…
