# Supplementary material for: The Wisdom Acquired During Emergencies Scale – Development and Validity
Source: Front Psychol. 2021 Oct 7;12:713404. doi: 10.3389/fpsyg.2021.713404 (PMC8530154; doi:10.3389/fpsyg.2021.713404)
Supplement: Supplementary file 2 [file Table_2.pdf]

Supplement 2

Frequencies and weights of the WADES items.

Options are sorted according to the weights (low = unwise, high= wise). The most left-hand number is the order of presentation during administration.

	frequency	weight
**#1 I'll be happy when…**		
5 People will admit it was all a joke	26	-1.780
6 I'll be able to escape from this house or this city	282	-1.203
7 I'll be free to do whatever I wish	1,141	-0.697
2 Those who are not law-abiding will be punished	103	-0.667
3 Those who are responsible for the pandemic will be punished	104	-0.657
4 I'll no longer have to be alone	177	-0.622
1 A vaccine will be found and infections will diminish	4,127	0.338
**#2 I'm convinced that most people…**		
4 Deliberately infect others	15	-1.316
7 Are going insane	639	-0.931
3 Believe that this is a conspiracy	397	-0.343
5 Don't care about the regulations	1,153	-0.143
2 Have negative and distressing thoughts	1,165	-0.112
1 Think that this situation is difficult but bearable	2,390	0.378
6 Help those who are in need	200	0.407
**#3 I'm afraid that people…**		
3 I don't care about people	91	-2.475
6 I don't think anything at all	313	-0.989
4 Will hurt animals	109	-0.842
7 I have no fears	272	-0.530
2 Are going to start breaking the law and will be dangerous	485	-0.468
5 Will continue to get ill	1,155	0.149
1 Haven't understood how serious the situation is	3,508	0.222
**#4 Above all, during this period what's needed is…**		
6 Being able to vent and distract oneself	294	-1.276
1 Giving in to selfishness, while staying at home, ignoring the outside world	188	-0.705
5 Having strong points of reference	1,077	-0.147
2 Frightening people so that they understand how serious the situation is	429	-0.114
7 Solidarity	444	0.081
4 Knowing that solutions will be found	1,854	0.156
3 Empathy, emotional support and reassurance	1,714	0.204
**#5 For kids, being isolated with their parents…**		
5 Can be sheer torture	175	-1.517
4 Is the most stressing thing at the moment	166	-1.423
2 Is a problem, because they miss their peers	333	-0.382
6 Has its pros and cons	2,675	-0.119
3 Is positive, if the relationships are positive	1,399	0.130
7 Is great	177	0.508
1 Means having a chance to get to know each other more	909	0.644

**#6 For those who have children, being quarantined…**		
7 Is exhausting	195	-1.488
5 Interferes with work	90	-0.985
3 Increases stress and tensions as well as the ability to communicate	1,678	-0.213
4 Is a way of getting to know one's children better	655	0.037
6 Allows them to relish being with them	856	0.110
2 Is a chance to teach them how to cope with a crisis	523	0.313
1 Allows them to share thoughts and rediscover values	1,183	0.528
**#7 At the moment I think beliefs…**		
7 Are misleading and deceptive	951	-0.783
4 Are exactly the same as before	1,860	-0.129
6 Can save us from this situation	73	0.085
3 Are stirred by the current difficulties	756	0.175
5 Are the only way to face the current situation	113	0.201
2 Are diminishing the distance between religions	246	0.325
1 Are helpful if they refer to ethical principles and science	1,827	0.385
**#8 To those who lost a loved one in this situation, I would like to say that…**		
4 You need to see a psychic	17	-1.877
3 The loss of your loved one is just one among many other losses	86	-1.822
2 The person who died is a victim of our politicians' incompetence	201	-0.790
6 I offer you my condolences	1,735	-0.445
5 Time heals all wounds	467	-0.394
7 I'll pray for him	475	0.363
1 We need to share the pain and memories	2,936	0.382
**#9 At bed-time…**		
6 I feel overwhelmed by anxiety	194	-0.802
7 I spend a lot of time online	1,241	-0.701
1 I wonder for how long I'll be able to bear this situation	400	-0.694
3 I constantly think about the current situation	177	-0.004
4 I think that sooner or later this situation will end	1,418	0.223
2 I prepare for the next day, setting myself some goals	1,888	0.369
5 I feel better thinking about my loved ones	546	0.502
**#10 The feelings I have had more often are…**		
6 Frustration	347	-1.213
5 A sense of emptiness	281	-1.101
7 Distress	219	-0.458
4 A sense of uncertainty	808	-0.066
1 Alternatingly positive and negative	2,519	0.026
2 Concerns about the health of my loved ones	1,157	0.472
3 A sense of solidarity and communion with others	593	0.515
**#11 When I think of the current situation, I feel…**		
7 Bored	283	-1.478
4 Abandoned	80	-1.058
5 Angry	299	-1.006
6 Confused	807	-0.346
3 Helpless and vulnerable	871	-0.176
2 Thankful that my loved ones are in good health	1,789	0.268
1 Hopeful that this health crisis will be resolved	1,842	0.400

**#12 The most unacceptable behaviours I heard about were…**		
2 Extremely controlling behaviours on behalf of the police force	298	-1.039
4 The idealisation of health workers	88	-0.922
7 Exaggerated patriotism	454	-0.538
1 Overreactions to coughing or sneezing	339	-0.449
5 The raiding of supermarkets	1,498	-0.035
3 Moaning about the restrictions we are subjected to	1,559	0.164
6 People underestimating the epidemic	1,751	0.313
**#13 During this period, what makes me feel better is…**		
5 Not hearing about the epidemic	340	-1.071
4 Spending time doing my favourite things	1,149	-0.468
6 Distracting myself with work	406	-0.432
1 Painstakingly disinfecting myself and my house	119	0.091
2 Thinking that my loved ones have not been infected by the virus	405	0.182
7 Helping others	289	0.202
3 Knowing that my loved ones are fine	3,245	0.282
**#14 The thought that I find most distressing during this period is…**		
5 That the quarantine will make us go insane	370	-1.026
2 That compared to the past, in the future there will be less freedom	1,931	-0.280
4 That the virus will destroy mankind	142	-0.083
7 That I could die all alone	228	-0.050
1 That the virus is nature’s revenge on the destructiveness of humankind	586	0.115
6 That I could infect other people	566	0.235
3 That my loved ones or I can get ill	1,941	0.376
**#15 The best thing about having to stay home is…**		
4 Being able to avoid the people we dislike	143	-1.301
7 None	487	-1.172
6 Being able to rest	341	-0.813
1 The testing of one’s frustration tolerance	489	-0.239
5 Being master of my time again	1,647	-0.118
3 Being able to dedicate more time to the people we love	923	0.452
2 Realising what is really important in life	1,948	0.458
**#16 I'm reassured by the fact that…**		
2 There is nothing that I find reassuring in this situation	219	-1.029
6 I have already lived most of my life	28	-0.974
3 I'm no longer the only person who senses hidden dangers	94	-0.697
5 This situation is difficult for everyone	871	-0.377
7 I'm reacting a lot better than I expected	1,017	-0.317
4 People who care about me are close by	976	0.041
1 My family and I are adopting all precautionary measures	2,698	0.365
**#17 I would like the people I live with to…**		
5 Leave me alone	255	-1.742
4 Stop making me feel how distressed they are	296	-0.966
6 Be more attentive to me	94	-0.577
7 Do nothing because I live alone	348	-0.304
3 Cooperate to a greater extent in household chores	389	-0.258
2 Be able to face this crisis in a positive way	898	0.113
1 Continue behaving responsibly, like they have done up to now	3,620	0.252

**#18 I'm very sad when I think…**		
3 That I don't really mind this situation	100	-1.415
1 That young people can't go out because of the elderly	85	-1.240
4 That I can't take advantage of the beautiful weather	343	-1.175
6 About the financial loss	675	-0.722
7 About those who are all alone	474	-0.187
5 That the ill are dying all alone in isolation	1,737	0.090
2 About the victims of the epidemic and their family and friends	2,551	0.426
**#19 During this crisis I ended up…**		
7 About wanting to die	75	-1.940
1 Thinking that for some people death could be a deliverance	206	-0.846
2 Thinking that I need some psychological support	503	-0.613
4 That humankind may become extinct	263	-0.421
6 About feeling vulnerable and fragile	1,360	-0.001
3 Thinking about making a substantial donation for the first time in my life	309	0.128
5 About reconsidering my priorities in life	2,858	0.294
**#20 In order to calm myself down, I…**		
2 Help myself with substances (alcohol, tablets, tobacco, or by overeating)	218	-1.242
3 I do whatever I feel like, when I feel like it	337	-0.885
6 I try not to think about anything	614	-0.561
5 I live in the here and now	736	-0.008
1 Spend my time engaging in constructive activities (sport, cooking, arts and crafts)	3,484	0.222
4 I talk to others about my concerns	363	0.229
7 I pray or meditate	165	0.319
**#21 The most positive aspect of this period is…**		
5 Being able to sleep or do absolutely nothing	176	-1.429
3 That I'm not forced into seeing people	196	-1.195
2 There is nothing positive about this nightmare	372	-1.167
6 Rediscovering what I'm passionate about	518	-0.288
7 That infections are decreasing	1,217	-0.198
4 Spending more time with my family	1,029	0.287
1 Rediscovering values like the solidarity among people	2,381	0.438
**#22 The most useful thing I have done…**		
7 Is sleeping, to avoid thinking	104	-1.659
3 I'm incapable of being useful	243	-1.347
4 Is not listening to the news	322	-0.872
1 Is being on my own, avoiding all contact with family and friends	986	-0.121
2 Is constantly listening to the news and checking the number of infected	325	-0.112
6 Is helping people in need	745	0.189
5 Is being law-abiding and making sure that others are too	3,114	0.264
**#23 When I think of my neighbours…**		
4 I wish they would mind their own business	166	-0.886
6 I never think about them	973	-0.785
2 I wish they were less noisy	447	-0.292
3 I wish they'd be more sociable	414	-0.115
7 I don't have neighbours	189	-0.077
1 I worry that they could be a source of infection	342	0.129
5 I hope they are well	3,415	0.301

**#24 Apart from following the regulations established by authorities, it would be helpful if one could…**		
3 Pretend the virus never existed	32	-2.027
5 Find those who are responsible for the epidemic	67	-1.232
7 No longer listen to the news	258	-1.066
2 Ignore the difficulties and avoid the drama	245	-0.862
4 Engage in different activities and keep busy	1,897	-0.211
6 Strengthen one’s religious beliefs	67	0.189
1 Consider the positive sides of difficult situations	3,343	0.305
**#25 This period will teach us...**		
7 That no one can save us	34	-1.755
5 That one can't trust people	47	-1.477
3 Nothing at all, people don't change	1,351	-0.749
4 That the world isn't a safe place	71	-0.450
6 To show more solidarity and be less selfish	752	0.146
2 To be more conscientious and have more common sense	1,715	0.261
1 To recognise our limits and that we are not invincible	1,935	0.326

The rationale of optimal scoring

The computation method of optimal scores is rather complex and the interested reader can find a thorough illustration in an introductory volume on correspondence analysis (e.g., Greenacre, 2007). For the purposes of our study, considering its unidimensional approach, with a single factor to be extracted, we can summarise the computational procedure as follows.

1. At first, for each answer, we chose one option as typical of situational wisdom. For the sake of simplicity, let us suppose that for each question the wisest answers are indicated by the letter A.
2. A unitary score is assigned to the wise answer, and zero to all others.
3. Compute the total score of the questionnaire for each participant.
4. For question 1. which has (for instance) five options as answers, compute the mean of the total score for all people who chose option A. Then the mean for those who chose option B and so forth.
5. Repeat the computations for all items.
6. Consider the computed mean of option A in item 1 as a better score to be assigned to option A in item 1.
7. Repeat the re-assignment for all the options, across all items.
8. Restart the procedure from point 4, and compute new scores for all options and all items, until no changes occur in the values.

After stabilisation occurs, high wisdom options will differ from low wisdom options: very wise people chose wise options and unwise individuals chose unwise options. In order to avoid unacceptable solutions (all zeros or infinite values), we must apply two constraints: the first one is to set the mean of the scores at zero. The second constraint must be applied to avoid null variance and therefore the variance is set to one. It should be noted that no matter what the initial values were, the iteration will always arrive at the same stabilised solution.

The data files are available here:

https://osf.io/rgw4t
