# Supplementary material for: The Wisdom Acquired During Emergencies Scale – Development and Validity
Source: Front Psychol. 2021 Oct 7;12:713404. doi: 10.3389/fpsyg.2021.713404 (PMC8530154; doi:10.3389/fpsyg.2021.713404)
Supplement: Supplementary file 3 [file Table_3.pdf]

Supplement 3: Conversion into percentiles

Wades

percentile	*N*	Mean	Min	Max
1	61	-3,742	-5,070	-3,080
2	62	-2,852	-3,060	-2,690
3	62	-2,505	-2,680	-2,380
4	62	-2,258	-2,360	-2,130
5	61	-2,038	-2,130	-1,970
6	62	-1,886	-1,970	-1,810
7	62	-1,753	-1,800	-1,700
8	62	-1,644	-1,700	-1,590
9	62	-1,537	-1,590	-1,490
10	61	-1,433	-1,480	-1,400
11	62	-1,341	-1,390	-1,300
12	62	-1,255	-1,300	-1,210
13	62	-1,168	-1,200	-1,130
14	62	-1,089	-1,130	-1,050
15	61	-1,014	-1,040	-0,980
16	62	-0,952	-0,980	-0,920
17	62	-0,892	-0,920	-0,870
18	62	-0,845	-0,870	-0,820
19	61	-0,791	-0,820	-0,760
20	62	-0,740	-0,760	-0,720
21	62	-0,697	-0,720	-0,680
22	62	-0,655	-0,680	-0,630
23	62	-0,608	-0,630	-0,580
24	61	-0,564	-0,580	-0,550
25	62	-0,528	-0,550	-0,510
26	62	-0,496	-0,510	-0,480
27	62	-0,460	-0,480	-0,440
28	62	-0,428	-0,440	-0,410
29	61	-0,395	-0,410	-0,380
30	62	-0,359	-0,380	-0,340
31	62	-0,327	-0,340	-0,310
32	62	-0,289	-0,310	-0,270
33	61	-0,256	-0,270	-0,240
34	62	-0,223	-0,240	-0,210
35	62	-0,194	-0,210	-0,180
36	62	-0,164	-0,180	-0,150

37	62	-0,133	-0,150	-0,120
38	61	-0,108	-0,120	-0,100
39	62	-0,082	-0,100	-0,070
40	62	-0,055	-0,070	-0,040
41	62	-0,023	-0,040	-0,010
42	62	0,003	-0,010	0,020
43	61	0,030	0,020	0,040
44	62	0,055	0,040	0,070
45	62	0,083	0,070	0,090
46	62	0,106	0,090	0,120
47	62	0,126	0,120	0,140
48	61	0,152	0,140	0,160
49	62	0,173	0,160	0,180
50	62	0,193	0,180	0,200
51	62	0,215	0,200	0,230
52	61	0,235	0,230	0,250
53	62	0,259	0,250	0,270
54	62	0,279	0,270	0,290
55	62	0,304	0,290	0,320
56	62	0,327	0,320	0,340
57	61	0,352	0,340	0,360
58	62	0,374	0,360	0,380
59	62	0,394	0,380	0,400
60	62	0,415	0,410	0,430
61	62	0,439	0,430	0,450
62	61	0,459	0,450	0,470
63	62	0,479	0,470	0,490
64	62	0,497	0,490	0,510
65	62	0,519	0,510	0,530
66	61	0,543	0,530	0,550
67	62	0,562	0,550	0,570
68	62	0,580	0,570	0,590
69	62	0,599	0,590	0,610
70	62	0,621	0,610	0,630
71	61	0,641	0,630	0,650
72	62	0,659	0,650	0,670
73	62	0,678	0,670	0,690
74	62	0,698	0,690	0,710
75	62	0,717	0,710	0,730
76	61	0,737	0,730	0,750

77	62	0,758	0,750	0,770
78	62	0,778	0,770	0,790
79	62	0,801	0,790	0,810
80	62	0,825	0,810	0,830
81	61	0,849	0,830	0,860
82	62	0,872	0,860	0,880
83	62	0,896	0,880	0,910
84	62	0,920	0,910	0,930
85	61	0,942	0,930	0,960
86	62	0,969	0,960	0,980
87	62	0,995	0,980	1,010
88	62	1,020	1,010	1,030
89	62	1,046	1,030	1,060
90	61	1,079	1,060	1,100
91	62	1,113	1,100	1,130
92	62	1,147	1,130	1,160
93	62	1,182	1,160	1,200
94	62	1,218	1,200	1,230
95	61	1,254	1,230	1,280
96	62	1,302	1,280	1,330
97	62	1,359	1,330	1,390
98	62	1,429	1,390	1,470
99	61	1,558	1,470	1,760
Total	6116	-0,010	-5,070	1,760
