# Supplementary material for: The Wisdom Acquired During Emergencies Scale – Development and Validity
Source: Front Psychol. 2021 Oct 7;12:713404. doi: 10.3389/fpsyg.2021.713404 (PMC8530154; doi:10.3389/fpsyg.2021.713404)
Supplement: Supplementary file 4 [file Table_4.pdf]

Supplement 4 Factor Matrix of the WADES

Note: The weights of supplement 2 have been applied to the whole sample (N=6116)

S01	0.474
S02	0.361
S03	0.421
S04	0.302
S05	0.385
S06	0.373
S07	0.353
S08	0.434
S09	0.433
S10	0.424
S11	0.459
S12	0.324
S13	0.364
S14	0.341
S15	0.494
S16	0.350
S17	0.424
S18	0.481
S19	0.370
S20	0.364
S21	0.505
S22	0.413
S23	0.380
S24	0.387
S25	0.428
